# Kaposi disease revealing HIV treatment breach: A report of two cases

**DOI:** 10.1002/ccr3.9253

**Published:** 2024-08-07

**Authors:** Camille Attal, Benjamin Demoury, Pierre Reimbold, Aurore Mensah, Géraldine Lescaille, Juliette Rochefort

**Affiliations:** ^1^ Oral and Dental Medicine Department Pitié Salpetrière Hospital, Paris Cité University Paris France; ^2^ Anatomy and Pathology Department Sorbonne University Paris France; ^3^ IHP group Laboratoire D'anatomie Pathologique IHP Paris Paris France

**Keywords:** AIDS, HIV, Kaposi disease, sexually transmitted disease

## Abstract

**Key Clinical Message:**

The purpose of this article is to highlight that oral Kaposi's disease can be indicative of a high viral load of HIV, either in the case of primary infection or therapeutic failure.

**Abstract:**

We report two cases of Kaposi Disease associated with HIV. The first case was a 30‐year‐old patient who unaware of her HIV‐positive status, and who was diagnosed with AIDS stage because of the biopsy revealed a gingival location of Kaposi disease. The second case was a 34‐year‐old patient who was referred to our department with a history of palatal lesion and claimed at first having no previous known medical conditions although his overall health condition seemed deteriorated. Our clinical examination was evocative of Kaposi Disease, which was confirmed by an emergency blood assessment and histological examination. Our diagnosis led us to disclose the HIV‐positive status of the patient and identify a progression to the AIDS stage, which allowed us to reintroduce the patient in the hospital framework. This case emphasizes the role of the oral surgeon as a key actor thanks to their knowledge of the clinical buccal manifestations of sexually transmitted infections (STI), in an era of resurgence of those conditions in vulnerable key populations.

## Graphical abstract

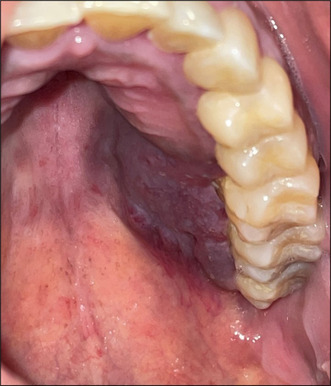

## 1 INTRODUCTION

Kaposi disease (KD) is a cutaneous, mucosal, and visceral neoplasia of endothelial origin, always associated with Human herpes virus‐8 (HHV8), also known as Kaposi's sarcoma‐associated Herpesvirus (KSHV). KSHV can be transmitted through saliva, sexual relations, vertical transmission, and contaminated blood transfusion. Its prevalence varies greatly worldwide: it is estimated that 50% of the Sub‐Saharian African population is infected with the virus, versus around 10% of the Western European, North American, and Asian population.^1^

Although the classical form described in 1872 by Moritz Kaposi mostly involved elderly men in the Mediterranean region, HIV‐related Kaposi Disease (HIV‐KD), individualized in the early 1980s, has become the prevalent form nowadays and is most frequently found among young men having sex with men (MSM).^2^ Additional variations of this disease include African endemic KD, described in the 1930s, and iatrogenic KD in patients receiving immunosuppressive therapy, particularly as a part of organ transplantations.^3^ In addition to those four etiopathogenic forms, the existence of a fifth subtype of KD, found in HIV‐negative MSM, has been recently suggested.^4^

The typical clinical manifestation is that of red to purple macules, papules, or plaques whose preferred anatomical localization depends on the epidemiological form of KD. It is estimated that 70% of patients diagnosed with HIV‐KD show an involvement of the oral cavity, those oral lesions being the first sign of the disease in around 20% of the cases.^5^

In some cases, oral lesions can serve to diagnose primary HIV infection or anti‐HIV therapeutic failure. We would like to describe two such cases, that were recently diagnosed in our department.

## 2 CASES PRESENTATION

Case number 1: diagnosis of primary HIV infection in oral lesion.

### 2.1 Case History/examination

**FIGURE 1:**
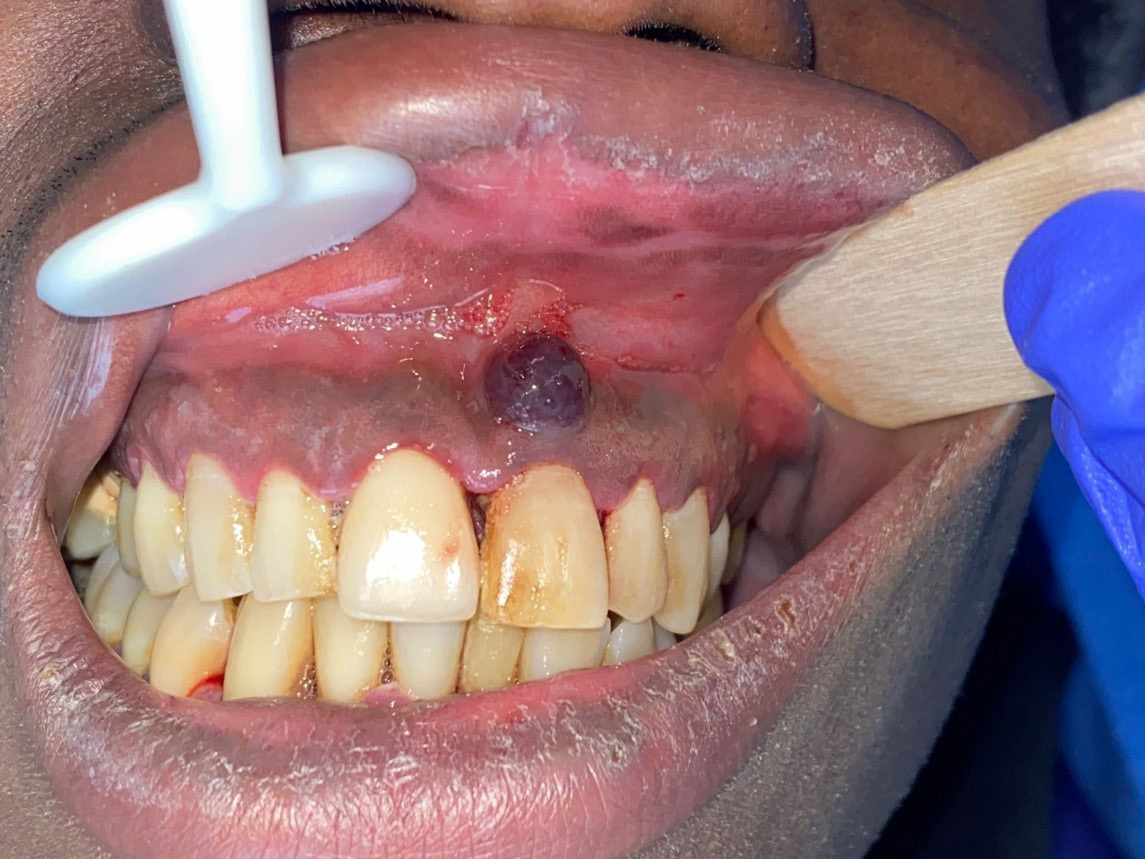
Gingival violaceous tumoral lesion of 1 cm, asymptomatic.

A 30‐year‐old patient, in a process of male‐to‐female gender transition, visited our department for acute gingival pain that had started 2 weeks ago, with an asymptomatic gingival violaceous tumoral lesion of 1 cm, (Figure 1). The patient, who suffered malnutrition, was hospitalized.

### 2.2 Methods (Differential diagnosis, investigations and treatment)

**FIGURE 2:**
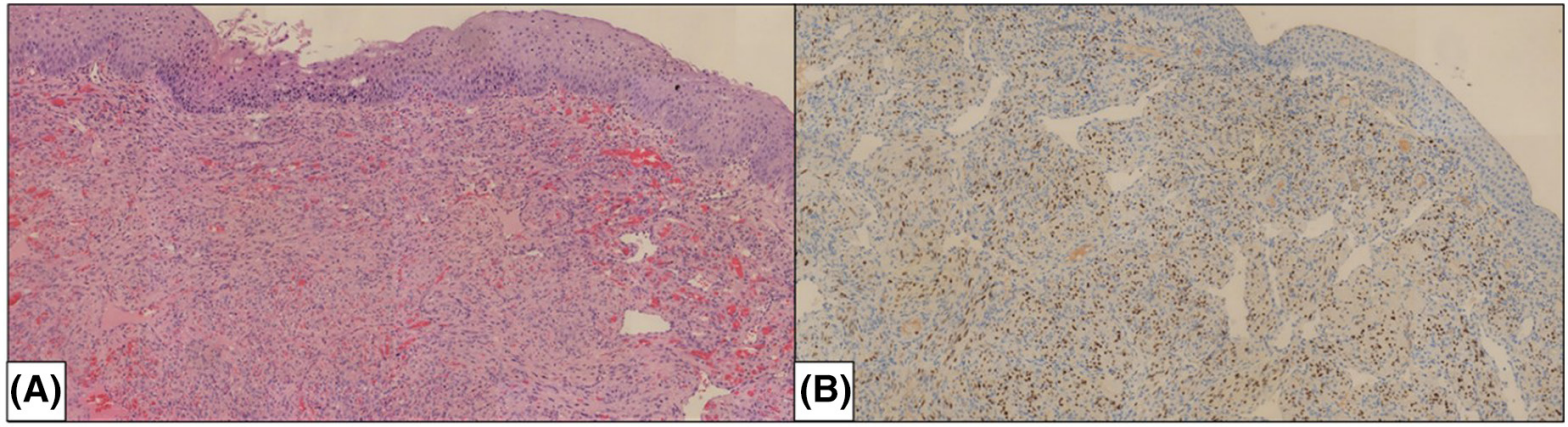
Magnification ×100. (A) HES staining with extravasated red blood cells stretched in slit‐like spaces, spindle cells with little atypicality, and siderophagous cells; (B) Positive IHC staining for HHV8.

The differential diagnoses to consider included a hemorrhagic blister or a hematoma, but the firm consistency of the lesion allowed these diagnostic options to be ruled out. Yet unaware of her HIV‐positive status, she was diagnosed with AIDS stage and the biopsy revealed a gingival location of KD (Figure 2).

Antiretroviral tritherapy was immediately initiated with Bictegravir/Emtricitabine/Tenofovir alafenamide, a single‐tablet regimen associating one Integrase inhibitor (INI) and two Nucleoside reverse transcriptase inhibitors (NRTI) which was granted its marketing authorization in Europe in 2018 for the treatment of HIV‐1 infection in treatment‐naive or virologically controlled pre‐treated patients, in the absence of INI, emtricitabine or tenofovir resistance.^6^

Case number 2: diagnosis of anti‐HIV therapeutic breakthrough in oral lesion.

### 2.3 Case history/examination

A 34‐year‐old man was referred to our service by his dentist for an asymptomatic palatal lesion that had been developing for several months and slowly increasing in size. The lump had originally started as a small nodule on the patient's palate and had been diagnosed by a previous dentist as a dental plaque‐related periodontitis.

**FIGURE 3:**
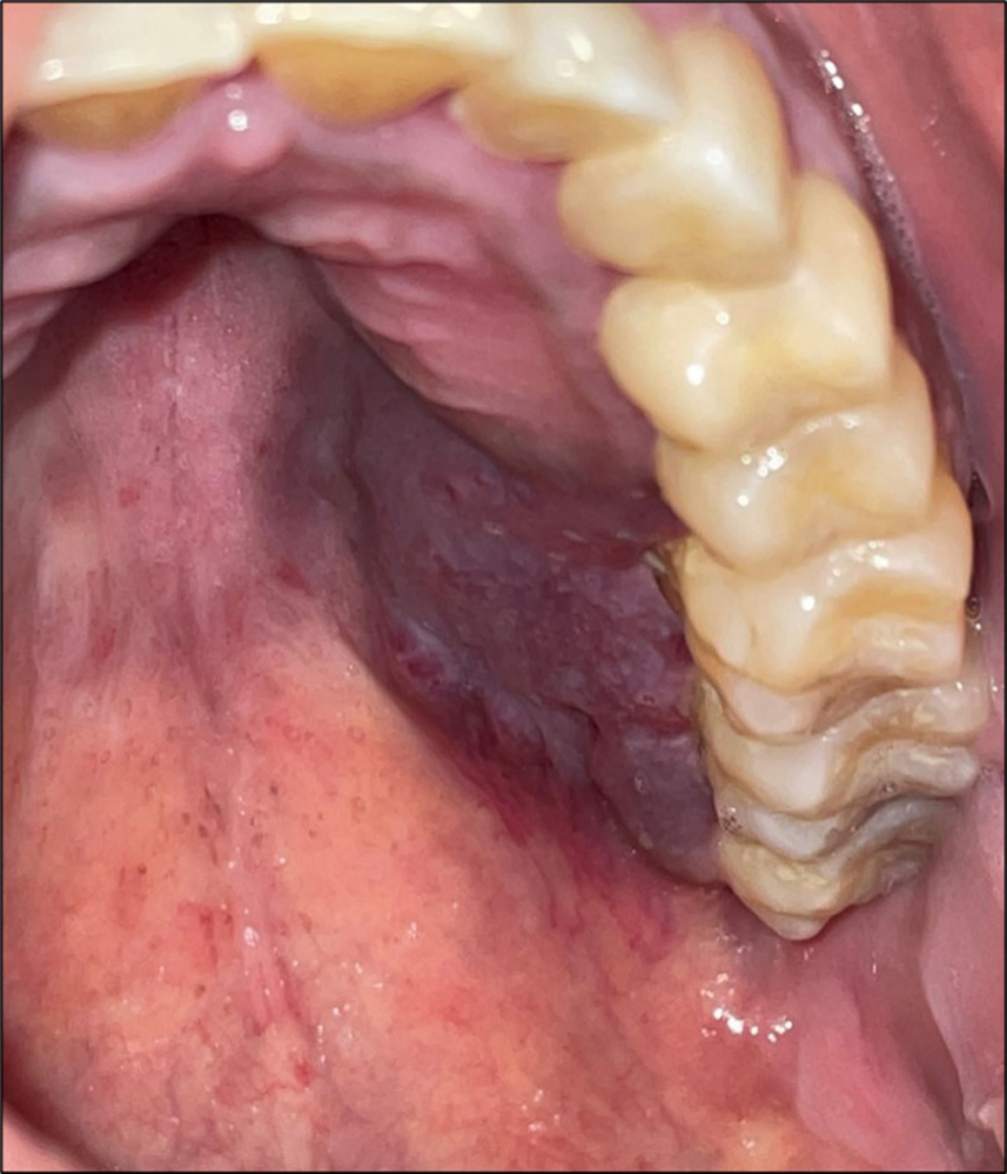
Palatal violaceous lesion of 3 cm, asymptomatic, with a detachment of the palatal mucosa, and non‐induration.

The patient was noted to have a seriously impaired general health condition with extreme thinness and asthenia and appeared nervous and hectic. He reported increased sleep disorders and feeding difficulties. The clinical examination, which was complicated by several episodes of emesis during the consultation, showed a thick purplish plaque of 3 cm along its longer axis on the left palatal mucosa, with a soft consistency and causing loss of adhesion of the fibro mucosa (Figure 3). He denied at first having any medical conditions, then admitted upon our insistence being HIV‐positive.

### 2.4 Methods (Differential diagnosis, investigations, and treatment)

**FIGURE 4:**
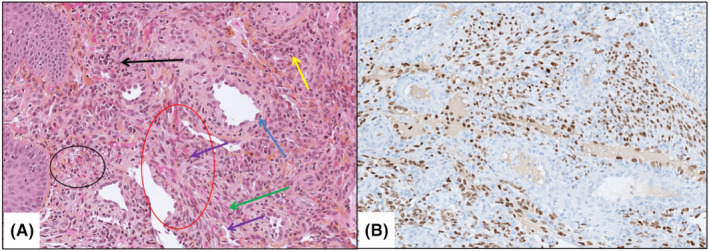
Magnification ×200. (A) HES staining. Black circle: Hyaline globules; Red circle: Extravasated red blood cells stretched in slit‐like spaces; Blue arrow: Slightly atypical turgid endothelial cells; Green arrow: Spindle cells with little atypicality; Purple arrows: Siderophagous cells; Yellow arrow: Some plasma cells; Black arrow: Some lymphocytes. (B) Positive IHC staining for HHV8.

The differential diagnosis to consider was a palatal hematoma but an emergency blood assessment was performed and showed severe immunosuppression with a low CD4 count of 11/mL of blood. Indeed, when this subset of T lymphocytes is so diminished, it indicates severe immunosuppression. Pathological examination (Figure 4) then revealed a positive anti‐HHV8 immunostaining on the spindle cells and lining the vascular structures, hereby confirming our diagnosis of immunosuppression‐related KD in a context of HIV treatment breach.

Further investigation into the patient's medical file revealed that he had been diagnosed with HIV‐1 in 2012 and had discontinued his treatments due to conflicts with hospital staff. His medical history included depression and paranoid personality disorders with a fear of persecution. Furthermore, he had no family in France and was in a precarious economic and social situation marked by unemployment and isolation. We organized continuity of care and he accepted to be transferred and hospitalized into the Department of infectious diseases of the hospital despite his initial reluctance.

## 3 CONCLUSION AND RESULTS (OUTCOME AND FOLLOW‐UP)

For the first case, a PCR assay was performed on the admission of the patient to the hospital and detected 7.26 HHV8 log copies/mL. After HIV resistance testing, antiretroviral therapy was initiated again with an INI (Dolutegravir) and a Non‐nucleoside reverse transcriptase inhibitor (Emtricitabine/Tenofovir), leading to an increase of the CD4 count to 139/mL of blood (vs. 11/mL of blood at the time of the consultation) and a decrease of the HHV8 viral load to 5.95 log copies/mL a few weeks after the reintroduction of cART. Additional systemic chemotherapy was proposed to the patient because of the spread of the lesions to the duodenum. Psychological followed‐up was also offered in an attempt to control the patient's depression, anxiety and sleep disorders.

For the second case, a triple therapy has been implemented, allowing for the rise of CD4+ T lymphocytes. Monitoring is conducted in the infectious diseases department. After 4 months of treatment, the purplish appearance hasn't diminished, but the nodular aspect of the lesion has decreased, giving way to a plaque and then a purplish patch.

## 4 DISCUSSION

Kaposi Disease is considered an AIDS‐defining disease when developing in HIV patients. As in other opportunistic conditions, therapeutic management is essentially based on immune reconstitution through combined antiretroviral therapy (cART).^7^ In moderate and slowly progressive forms of KD, immune restoration is often sufficient to stabilize or heal KD lesions.^8^

Additional local and general treatments can also be provided, depending on the spread of the cutaneous and visceral lesions and the persistence of the disease in spite of the implementation of cART.^9^ Local therapies include surgery, local chemotherapy, immunotherapy, cryotherapy, and radiotherapy, and lead to fewer side effects and interactions with the patient's drugs than systemic treatments. Systemic chemotherapy should be conducted in case of aggressive progression of the disease, extensive mucosal involvement or severe accompanying symptoms such as hemorrhage and can be combined with local treatments.^8^

The incidence of KD has dramatically decreased in developed countries with the generalization of cART since its introduction in 1995, and the prognosis of the patients has greatly improved.^8^ However, data from the Joint United Nations Program on HIV/AIDS (UNAIDS) has showed a resurgence in the incidence of HIV infections over the last decade (2010–2019) in over 30 countries including the United States of America, Brazil, Spain, Portugal and Ukraine.^10^ Reasons for this rise remain unclear but the majority of these new infections occur among key populations which suffer low access to healthcare such as people with disabilities, low economic and social status, injecting drug users, sex workers and transgender people.^11^ New HIV infections also disproportionally occurs in young people (15–24 years), who represented 28% of new HIV infections in 2019 although they constitute about 15% of the global population. At the era of pre‐exposure prophylaxis (PrEP), which constitute a significant part of the UNAIDS's program to eradicate AIDS by 2030, these discrepancies emphasize the inequalities remaining between social groups in terms of healthcare access, even in developed countries.^12^

Proper and long‐term adherence of the patient to cART is essential to achieve viral suppression, but also to prevent the development of drug resistance by the selection of resistant mutations of the virus. The relationship between patient compliance and development of resistance is different for each antiretroviral drug.^13^

Because of the increasing threat of drug resistance on the efficiency of antiretroviral therapies, the World Health Organization (WHO) has replaced non‐nucleoside reverse transcriptase inhibitors (NNRTI) with Dolutegravir (DTG), a second‐generation INI, as the first‐line treatment recommendation in its 2018 guidelines.^14^ However, in spite of DTG's higher genetic barrier to resistance, some cases of emergent DTG resistance have been reported. Identified risk factors include low medication adherence, drug interactions, high baseline viral load and active opportunistic infections.^15^

Lenacapavir, the first HIV capsid inhibitor, was approved in 2022 in Europe and in the United States of America as a new drug to be used in addition to other antiretroviral treatments for heavily treatment‐experienced people with multiresistant HIV‐1 infection. Its mode of administration makes it particularly interesting for people who have undergone treatment failure due to suboptimal adherence, as it only requires two injections a year.^16^ Therefore, this treatment represents a new chance for patients whose vital prognosis and quality of life were jeopardized by the exhaustion of treatment options due to resistance mutations of the virus.

It has been suggested that social determinants such as non‐white race, low level of education, poverty and unemployment are associated with lower levels of compliance with cART treatment.^17^ These disparities could be explained by the barriers these groups of people have to overcome to access healthcare, including economic difficulties, racism, and fear of stigma. At the crossroads of multiple forms of discrimination, young Black MSM constitute a high‐risk group for lower levels of HIV testing and treatment.^18^

On the individual level, stress, post‐traumatic stress disorder (PTSD), and most importantly depression have also been identified as strong co‐factors of patient non‐adherence by several studies.^19^ Depressed HIV patients are more at risk of missed medical appointments, hereby displaying increased viral load and suffering overall higher mortality rates than non‐depressed patients. This observation emphasizes the necessity to identify and treat adequately depression among HIV patients.^20^ In this situation, healthcare professionals discovering treatment breaches play a crucial role in restoring the patient's trust in the medical staff and advocating for mental health management, which is closely linked with treatment success.

In this regard, oral cavity specialists have a decisive role to play on the vital prognosis thanks to their knowledge of clinical oral manifestations of opportunist infectious diseases, re‐engaging patients in a multidisciplinary framework of comprehensive care.

Indeed, many oral lesions are markers of immune deterioration as they arise from opportunistic conditions, and can therefore reveal a progression of HIV.^21^ Thus, major aphthous ulcers, necrotizing ulcerative periodontitis, intraoral Kaposi's disease, oral leukoplakia, and candidiasis have been found to have a predictive value of finding CD4+ cell counts below 200/mm^3^ in HIV patients.^22^ This can also be the case for atypical or secondary infected herpetic recurrences, as well as for multiple papilloma.^23^ During the last few months, we have noted an increase of those cases reported in our consultation centre (four patients in 3 months), although these are considered extremely rare in France nowadays.

Given the re‐emergence of sexually transmitted diseases during the last few years, especially among MSM,^24^ increasing the level of training of dentists and oral health providers about venereal diseases is of critical importance as an early diagnosis can improve the patients' vital prognosis. It is also important for healthcare professionals to be aware of the groups of patients who have a greater risk of non‐adherence to their treatments, and to advocate for mental health care as an integrant part of the patient's global follow‐up.

## AUTHOR CONTRIBUTIONS

**Camille Attal:** Data curation; formal analysis; writing – original draft. **Benjamin Demoury:** Data curation; writing – original draft. **Pierre Reimbold:** Formal analysis; writing – original draft. **Aurore Mensah:** Formal analysis; writing – original draft. **Géraldine Lescaille:** Project administration; writing – original draft. **Juliette Rochefort:** Conceptualization; formal analysis; methodology; project administration; supervision; validation; writing – original draft.

## FUNDING INFORMATION

None.

## CONFLICT OF INTEREST STATEMENT

All authors have no conflicts of interest to disclose.

## CONSENT

Written informed consent was obtained from the patient to publish this report in accordance with the journal's patient consent policy.

## Data Availability

The data that support the findings of this study are available on request from the corresponding author. The data are not publicly available due to privacy or ethical restrictions.
